# Supplementary material for: Breaking up classroom sitting time with cognitively engaging physical activity: Behavioural and brain responses
Source: PLoS One. 2021 Jul 14;16(7):e0253733. doi: 10.1371/journal.pone.0253733 (PMC8279315; doi:10.1371/journal.pone.0253733)
Supplement: S1 Checklist — (PDF) [file pone.0253733.s001.pdf]

TREND Statement Checklist

Paper Section/Topic	Item No	Descriptor	Reported? ✓	Reported? Pg #
**Title and Abstract**				
Title and Abstract	1	• Information on how units were allocated to interventions	✓	2
		• Structured abstract recommended	✓	2-3
		• Information on target population or study sample	✓	2
**Introduction**				
Background	2	• Scientific background and explanation of rationale	✓	5-10
		• Theories used in designing behavioral interventions	✓	5-8
**Methods**				
Participants	3	• Eligibility criteria for participants, including criteria at different levels in recruitment/sampling plan (e.g., cities, clinics, subjects)	✓	11
		• Method of recruitment (e.g., referral, self-selection), including the sampling method if a systematic sampling plan was implemented	✓	10-11
		• Recruitment setting	✓	10-11
		• Settings and locations where the data were collected	✓	13-19
Interventions	4	• Details of the interventions intended for each study condition and how and when they were actually administered, specifically including:	✓	12-13, S2 Table
		○ Content: what was given?	✓	12-13, S2 Table
		○ Delivery method: how was the content given?	✓	12-13, S2 Table
		○ Unit of delivery: how were subjects grouped during delivery?	✓	12
		○ Deliverer: who delivered the intervention?	✓	12
		○ Setting: where was the intervention delivered?	✓	12
		○ Exposure quantity and duration: how many sessions or episodes or events were intended to be delivered? How long were they intended to last?	✓	12
		○ Time span: how long was it intended to take to deliver the intervention to each unit?	✓	12
		○ Activities to increase compliance or adherence (e.g., incentives)	✓	12-13
Objectives	5	• Specific objectives and hypotheses	✓	9-10
Outcomes	6	• Clearly defined primary and secondary outcome measures	✓	14-19
		• Methods used to collect data and any methods used to enhance the quality of measurements	✓	14-19
		• Information on validated instruments such as psychometric and biometric properties	✓	14-19
Sample size	7	• How sample size was determined and, when applicable, explanation of any interim analyses and stopping rules	✓	11
Assignment method	8	• Unit of assignment (the unit being assigned to study condition, e.g., individual, group, community)	✓	12
		• Method used to assign units to study conditions, including details of any restriction (e.g., blocking, stratification, minimization)	✓	12
		• Inclusion of aspects employed to help minimize potential bias induced due to non-randomization (e.g., matching)	✓	12
Blinding (masking)	9	• Whether or not participants, those administering the interventions, and those assessing the outcomes were blinded to study condition assignment; if so, statement regarding how the blinding was accomplished and how it was assessed		n/a
Unit of Analysis	10	• Description of the smallest unit that is being analysed to assess intervention effects (e.g., individual, group, or community)	✓	13-21
		• If the unit of analysis differs from the unit of assignment, the analytical method used to account for this (e.g., adjusting the standard error estimates by the design effect or	✓	19-22

TREND Statement Checklist

		using multilevel analysis)		
Statistical methods	11	• Statistical methods used to compare study groups for primary methods outcome(s), including complex methods for correlated data	✓	20
		• Statistical methods used for additional analyses, such as subgroup analyses and adjusted analysis	✓	19, 21-22
		• Methods for imputing missing data, if used		n/a
		• Statistical software or programs used	✓	14-17, 21-22
**Results**				
Participant flow	12	• Flow of participants through each stage of the study: enrollment, assignment, allocation and intervention exposure, follow-up, analysis (a diagram is strongly recommended)	✓	11, Fig 1
		○ Enrollment: the numbers of participants screened for eligibility, found to be eligible or not eligible, declined to be enrolled, and enrolled in the study	✓	11, 22, Fig 1
		○ Assignment: the numbers of participants assigned to a study condition	✓	11, Fig 1
		○ Allocation and intervention exposure: the number of participants assigned to each study condition and the number of participants who received each intervention	✓	11, Fig 1
		○ Follow-up: the number of participants who completed the follow-up or did not complete the follow-up (i.e., lost to follow-up), by study condition	✓	11, Fig 1
		○ Analysis: the number of participants included in or excluded from the main analysis, by study condition	✓	11, Fig 1
		• Description of protocol deviations from study as planned, along with reasons		n/a
Recruitment	13	• Dates defining the periods of recruitment and follow-up	✓	10, 13
Baseline data	14	• Baseline demographic and clinical characteristics of participants in each study condition	✓	22
		• Baseline characteristics for each study condition relevant to specific disease prevention research	✓	22
		• Baseline comparisons of those lost to follow-up and those retained, overall and by study condition		n/a
		• Comparison between study population at baseline and target population of interest		n/a
Baseline equivalence	15	• Data on study group equivalence at baseline and statistical methods used to control for baseline differences	✓	22-28
Numbers analyzed	16	• Number of participants (denominator) included in each analysis for each study condition, particularly when the denominators change for different outcomes; statement of the results in absolute numbers when feasible	✓	11, Fig 1, 22
		• Indication of whether the analysis strategy was “intention to treat” or, if not, description of how non-compliers were treated in the analyses	✓	11, 22-23
Outcomes and estimation	17	• For each primary and secondary outcome, a summary of results for each estimation study condition, and the estimated effect size and a confidence interval to indicate the precision	✓	22-29
		• Inclusion of null and negative findings	✓	22-29
		• Inclusion of results from testing pre-specified causal pathways through which the intervention was intended to operate, if any	✓	26-27
Ancillary analyses	18	• Summary of other analyses performed, including subgroup or restricted analyses, indicating which are pre-specified or exploratory	✓	25-29
Adverse events	19	• Summary of all important adverse events or unintended effects in each study condition (including summary measures, effect size estimates, and confidence intervals)	✓	28-29
**Discussion**				
Interpretation	20	• Interpretation of the results, taking into account study hypotheses, sources of potential bias, imprecision of measures, multiplicative analyses, and other limitations or weaknesses of the study	✓	29-37

TREND Statement Checklist

		• Discussion of results taking into account the mechanism by which the intervention was intended to work (causal pathways) or alternative mechanisms or explanations	✓	30-33
		• Discussion of the success of and barriers to implementing the intervention, fidelity of implementation	✓	33-34, 35-36
		• Discussion of research, programmatic, or policy implications	✓	33, 37
Generalizability	21	• Generalizability (external validity) of the trial findings, taking into account the study population, the characteristics of the intervention, length of follow-up, incentives, compliance rates, specific sites/settings involved in the study, and other contextual issues	✓	35-36
Overall evidence	22	• General interpretation of the results in the context of current evidence and current theory	✓	37

From: Des Jarlais, D. C., Lyles, C., Crepaz, N., & the Trend Group (2004). Improving the reporting quality of nonrandomized evaluations of behavioral and public health interventions: The TREND statement. *American Journal of Public Health*, 94, 361-366. For more information, visit: http://www.cdc.gov/trendstatement/
