# Supplementary material for: Breaking up classroom sitting time with cognitively engaging physical activity: Behavioural and brain responses
Source: PLoS One. 2021 Jul 14;16(7):e0253733. doi: 10.1371/journal.pone.0253733 (PMC8279315; doi:10.1371/journal.pone.0253733)
Supplement: S1 Table — (PDF) [file pone.0253733.s002.pdf]

S1 Table. Simple and cognitively engaging intervention activities.

Simple active breaks	Cognitively engaging active breaks
1) *Quick fit!* – a simple imitation of a movement sequence.<br>2) *Silent ball* – children try to toss a light ball to each other without talking, making sounds or dropping the ball.<br>3) *As if…* – children enact the actions described in the sentences read by the teacher.<br>4) *Fitness dice* – children perform the activity associated with the result from a dice roll.<br>5) *Over, Under, Around and Through* – children form lines of four/five and go over, under, around and through imaginary or real objects following their leader.<br>6) *3-speed car* – children pretend to be cars travelling at different speeds as suggested by the teacher.<br>7) *Let's dance!* – children dance for the duration of a fun music track.	1) *My Clock is Late!* – an imitation of a coordination sequence with a time delay between teacher and children [60].<br>2) *Silent ball Q&A* – children toss a ball to each other, each time the thrower asks a question to which the catcher has to answer.<br>3) *Simon says…* – children perform the actions that are preceded by 'Simon says…' but do nothing in absence of that phrase.<br>4) *Robot remote control* – a stimulus-response game that requires children remember and perform the appropriate positions/actions associated to each of the teacher's prompts.<br>5) *One, two, three…star (+ moon, + sun)* – a game that requires children to quickly respond to the stimuli provided by the curator of the game [60].<br>6) *Crazy traffic lights* – children are required to move or stop according to the visual signs presented by the teacher, disregarding the inconsistent verbal cues that the teacher will sometimes provide [60].<br>7) *Dance off!* – children dance over a music track and freeze their position every time the music stops.
