# Supplementary material for: Breaking up classroom sitting time with cognitively engaging physical activity: Behavioural and brain responses
Source: PLoS One. 2021 Jul 14;16(7):e0253733. doi: 10.1371/journal.pone.0253733 (PMC8279315; doi:10.1371/journal.pone.0253733)
Supplement: S2 Table — (PDF) [file pone.0253733.s003.pdf]

S2 Table. Haemodynamic response data processing pipeline and MATLAB processing script.

Process	Specific	Value	Description
HMR intensity to OD	n/a	n/a	The intensity data (number of time points multiplied by the number of data channels) are divided by the mean, and then converted to the change in OD.
HMR motion artefact remover by channel	Time motion window (s)<br>Time mask (s)<br>SD threshold<br>Amplitude threshold	0.5<br>1.0<br>50.0<br>0.10	These filters identify and remove motion artefacts. Segments of data channels that exhibit a change that are greater than: (i) the SD threshold multiplied by the SD of the intensity data, and/or (ii) the indicated amplitude threshold, within an indicated time range, are marked as artefacts for +/– the mask time.
HMR motion correction PCA filter	Number of principal components	0.97	This function uses PCA to filter the segments identified as motion artefacts, according to the number of principal components to remove from the data.
HMR band pass filter	High pass filter<br>Low pass filter	0.010<br>0.20	This perform bandpass filters: high pass filter frequency (Hz), typical values between 0 and 0.02; low pass filter frequency (Hz), typical values between 0.5 and 3.
HMR OD to concentrations	Partial pathlength factors	6.0<br>6.0	For each wavelength, partial pathlength factors are identified. Typical values are around 6.
HMR block average	Time range (s)	−5.0<br>30.0	This part of the process calculates the block average for each condition within the defined time range.

MATLAB processing script

```
dod = hmrIntensity2OD(d);
[tIncAuto,tIncChAuto] = hmrMotionArtifactByChannel(dod,t,SD,tIncMan,0.5,1,50,0.1);
[dod,svsMotion,nSVMotion] = hmrMotionCorrectPCA(SD,dod,tIncAuto,0.97);
dod = hmrBandpassFilt(dod,t,0.01,0.2);
dc = hmrOD2Conc(dod,SD,[6 6]);
[dcAvg,dcAvgStd,tHRF,nTrials,dcSum2] = hmrBlockAvg(dc,s,t,[−5 30]);
```

HMR, haemo-dynamic response; OD, optical density; SD, standard deviation; PCA, principal component analysis.
