# Supplementary material for: Breaking up classroom sitting time with cognitively engaging physical activity: Behavioural and brain responses
Source: PLoS One. 2021 Jul 14;16(7):e0253733. doi: 10.1371/journal.pone.0253733 (PMC8279315; doi:10.1371/journal.pone.0253733)
Supplement: S3 Table — All models were adjusted for the baseline value related to each outcome variable, child sex and age, and controlled for the random effects of classroom as a clustering variable. (PDF) [file pone.0253733.s004.pdf]

S3 Table. Results from linear mixed models, used to understand differences by study group on the change in sitting, standing, and stepping patterns.

Activity pattern	Mean Δ from baseline [95% CI]			Intervention effects (Mean Δ [95% CI])	
	Control	Simple active breaks	Cognitive engaging active breaks	Simple vs Control	Cognitively engaging vs Control
**Class time at mid-trial**					
n	40	46	50	–	–
Sitting (min)	–4.36<br>[–13.31, 4.58]	–5.89<br>[–14.95, 3.16]	7.77<br>[–0.80, 16.34]	–1.53<br>[–14.25, 11.20]	12.13<br>[–0.40, 24.67]
Sitting bouts > 5 min (freq)	0.14<br>[–0.95, 1.22]	–0.62<br>[–1.79, 0.54]	0.48<br>[–0.61, 1.56]	–0.76<br>[–2.35, 0.84]	0.34<br>[–1.21, 1.89]
Sitting bouts > 5 min (min)	–29.95***<br>[–46.45, –13.45]	–20.85*<br>[–38.71, –2.99]	5.75<br>[–10.74, 22.23]	9.10<br>[–15.28, 33.47]	35.69**<br>[12.20, 59.19]
Sitting bouts > 20 min (freq)	–0.68**<br>[–1.15, –0.21]	–0.35<br>[–0.86, 0.16]	–0.08<br>[–0.55, 0.39]	0.33<br>[–0.37, 1.03]	0.60<br>[–0.08, 1.28]
Sitting bouts > 20 min (min)	–32.95***<br>[–46.87, –19.02]	–16.54*<br>[–31.55, –1.53]	–4.14<br>[–17.96, 9.67]	16.41<br>[–4.15, 36.97]	28.80**<br>[9.12, 48.49]
Sit-to-stand transitions (freq)	8.66***<br>[5.48, 11.85]	6.75***<br>[3.48, 10.02]	–1.61<br>[–4.68, 1.45]	–1.92<br>[–6.47, 2.64]	–10.28***<br>[–14.71, –5.85]
Standing (min)	0.93<br>[–5.62, 7.47]	3.61<br>[–2.61, 9.83]	–6.25*<br>[–12.18, –0.32]	2.68<br>[–6.43, 11.80]	–7.18<br>[–16.14, 1.79]
Stepping (min)	3.50<br>[–0.18, 7.18]	1.84<br>[–2.26, 5.93]	–1.37<br>[–5.11, 2.38]	–1.66<br>[–7.19, 3.86]	–4.86<br>[–10.10, 0.37]
Total step count (freq)	215.68<br>[–59.41, 490.76]	103.20<br>[–204.44, 410.85]	–68.82<br>[–348.92, 211.28]	–112.48<br>[–528.03, 303.08]	–284.50<br>[–674.96, 105.95]
**School time at mid-trial**					
n	40	46	50	–	–
Sitting (min)	–5.40<br>[–15.54, 4.74]	–4.62<br>[–14.06, 4.81]	11.96*<br>[2.78, 21.14]	0.77<br>[–13.01, 14.55]	17.36*<br>[3.53, 31.20]
Sitting bouts > 5 min (freq)	0.36<br>[–0.84, 1.55]	–0.67<br>[–1.94, 0.59]	0.84<br>[–0.34, 2.02]	–1.03<br>[–2.77, 0.71]	0.49<br>[–1.20, 2.18]
Sitting bouts > 5 min (min)	–27.87***<br>[–43.45, –12.29]	–22.48**<br>[–38.04, –6.91]	10.11<br>[–4.62, 24.84]	5.39<br>[–16.67, 27.45]	37.98**<br>[16.31, 59.65]
Sitting bouts > 20 min (freq)	–0.72**<br>[–1.17, –0.27]	–0.35<br>[–0.83, 0.12]	–0.05<br>[–0.49, 0.39]	0.37<br>[–0.29, 1.03]	0.67*<br>[0.03, 1.31]

Sitting bouts > 20 min (min)	–33.05***<br>[–46.74, –19.35]	–17.33*<br>[–31.31, –3.35]	–2.70<br>[–15.76, 10.36]	15.72<br>[–3.95, 35.38]	30.34**<br>[11.31, 49.38]
Sit-to-stand transitions (freq)	8.69***<br>[5.08, 12.30]	4.87**<br>[1.23, 8.51]	–2.92<br>[–6.36, 0.51]	–3.82<br>[–8.94, 1.30]	–11.61***<br>[–16.62, –6.60]
Standing (min)	1.77<br>[–6.00, 9.55]	6.07<br>[–0.99, 13.13]	–4.18<br>[–11.05, 2.70]	4.30<br>[–6.24, 14.84]	–5.95<br>[–16.55, 4.65]
Stepping (min)	3.34<br>[–1.63, 8.30]	–1.89<br>[–6.60, 2.81]	–7.11**<br>[–11.55, –2.67]	–5.23<br>[–12.15, 1.69]	–10.44**<br>[–17.07, –3.82]
Total step count (freq)	164.48<br>[–245.09, 574.05]	–190.32<br>[–575.28, 194.63]	–528.96**<br>[–889.58, –168.35]	–354.80<br>[–928.34, 218.73]	–693.44*<br>[–1236.96, –149.93]
**Class time at end of trial**					
n	40	47	50	–	–
Sitting (min)	10.98*<br>[1.99, 19.97]	–2.56<br>[–12.03, 6.91]	–2.49<br>[–11.37, 6.39]	–13.53*<br>[–26.59, –0.48]	–13.47*<br>[–26.22, –0.71]
Sitting bouts > 5 min (freq)	–0.17<br>[–0.99, 0.65]	–0.51<br>[–1.36, 0.34]	–0.05<br>[–0.85, 0.76]	–0.34<br>[–1.53, 0.84]	0.12<br>[–1.04, 1.29]
Sitting bouts > 5 min (min)	12.42<br>[–1.41, 26.25]	–8.06<br>[–22.49, 6.37]	–3.94<br>[–17.46, 9.57]	–20.48*<br>[–40.50, –0.47]	–16.36<br>[–35.91, 3.19]
Sitting bouts > 20 min (freq)	0.26<br>[–0.10, 0.63]	–0.22<br>[–0.59, 0.15]	–0.03<br>[–0.38, 0.32]	–0.49<br>[–1.01, 0.04]	–0.29<br>[–0.81, 0.23]
Sitting bouts > 20 min (min)	15.36*<br>[3.00, 27.71]	–8.58<br>[–21.39, 4.23]	–3.57<br>[–15.53, 8.39]	–23.94**<br>[–41.79, –6.09]	–18.93*<br>[–36.22, –1.63]
Sit-to-stand transitions (freq)	3.02*<br>[0.01, 6.02]	4.59**<br>[1.68, 7.50]	3.33*<br>[0.53, 6.13]	1.57<br>[–2.60, 5.74]	0.32<br>[–3.81, 4.44]
Standing (min)	–8.96*<br>[–17.14, –0.79]	2.98<br>[–5.86, 11.82]	–0.23<br>[–8.40, 7.94]	11.94<br>[–0.13, 24.02]	8.73<br>[–2.90, 20.36]
Stepping (min)	–1.43<br>[–3.69, 0.83]	–0.35<br>[–2.68, 1.97]	2.35*<br>[0.16, 4.55]	1.08<br>[–2.18, 4.33]	3.78*<br>[0.64, 6.92]
Total step count (freq)	–92.09<br>[–236.59, 52.40]	–64.30<br>[–209.68, 81.09]	172.76*<br>[35.64, 309.89]	27.80<br>[–179.15, 234.75]	264.86**<br>[67.16, 462.56]
**School time at end of trial**					
n	40	47	50	–	–
Sitting (min)	12.23**<br>[3.22, 21.25]	–1.25<br>[–9.74, 7.24]	–0.99<br>[–9.30, 7.32]	–13.49*<br>[–25.79, –1.18]	–13.22*<br>[–25.63, –0.82]
Sitting bouts > 5 min (freq)	–0.47<br>[–1.27, 0.33]	–0.63<br>[–1.41, 0.14]	0.13<br>[–0.61, 0.88]	–0.16<br>[–1.27, 0.95]	0.60<br>[–0.50, 1.71]

Sitting bouts > 5 min (min)	15.51*<br>[2.87, 28.15]	–11.24<br>[–23.37, 0.89]	–4.42<br>[–16.15, 7.30]	–26.75**<br>[–44.26, –9.24]	–19.94*<br>[–37.45, –2.42]
Sitting bouts > 20 min (freq)	0.32<br>[–0.04, 0.69]	–0.31<br>[–0.68, 0.05]	–0.07<br>[–0.41, 0.28]	–0.64*<br>[–1.15, –0.12]	–0.39<br>[–0.91, 0.12]
Sitting bouts > 20 min (min)	21.70***<br>[9.91, 33.49]	–12.33*<br>[–24.07, –0.59]	–5.68<br>[–16.79, 5.44]	–34.03***<br>[–50.72, –17.34]	–27.38***<br>[–43.72, –11.03]
Sit-to-stand transitions (freq)	2.86<br>[–0.73, 6.45]	3.79*<br>[0.39, 7.18]	1.28<br>[–2.02, 4.58]	0.93<br>[–3.99, 5.84]	–1.58<br>[–6.50, 3.34]
Standing (min)	–8.06<br>[–17.85, 1.72]	3.91<br>[–6.73, 14.56]	–0.19<br>[–10.03, 9.65]	11.98<br>[–2.50, 26.46]	7.88<br>[–6.10, 21.86]
Stepping (min)	–5.14<br>[–10.95, 0.67]	–0.98<br>[–7.20, 5.24]	1.36<br>[–4.36, 7.09]	4.16<br>[–4.42, 12.74]	6.50<br>[–1.62, 14.63]
Total step count (freq)	–454.02<br>[–933.48, 25.43]	–225.34<br>[–737.43, 286.75]	91.66<br>[–377.89, 561.20]	228.68<br>[–481.11, 938.47]	545.68<br>[–122.64, 1214.00]

CI, confidence interval. All models were adjusted for the baseline value related to each outcome variable, child sex and age, and controlled for the random effects of classroom as a clustering variable.

*$p < 0.05$; **$p < 0.01$; ***$p < 0.001$.
