# Supplementary material for: Breaking up classroom sitting time with cognitively engaging physical activity: Behavioural and brain responses
Source: PLoS One. 2021 Jul 14;16(7):e0253733. doi: 10.1371/journal.pone.0253733 (PMC8279315; doi:10.1371/journal.pone.0253733)
Supplement: S1 Data — (PDF) [file pone.0253733.s005.pdf]

Trial study protocol

The present document reflects the protocol as approved by the ethics committee prior the start of the trial. If you desire to confirm that the document submitted conforms with what was approved by the ethics committee you may contact the Human Research Ethics Office, Deakin University, 221 Burwood Highway, Burwood Victoria 3125, Telephone: +61 3 9251 7129, research-ethics@deakin.edu.au (please quote project number 2016-382).

Background, aim, and hypothesis

Increasing children's physical activity is an important public health priority. The health-benefits of physical activity are well documented and growing evidence shows that being physically active may include improvements in cognitive functions [1]. On the other hand, there is evidence that links sedentary behaviour to poorer physical health in children, independent from physical activity levels [2]. A few classroom-based strategies aimed at improving physical activity and reducing sedentary behaviour (e.g., activity breaks) have recently been tested in schoolchildren. Overall, these strategies seem to improve children's health and may also benefit some cognitive outcomes [3]. Cognitively engaging types of physical activity are hypothesized to benefit cognitive functions more than simple physical activity types [1]. However, thus far only three studies [4–6] have investigated the effects of cognitively engaging types of active breaks on cognitive functions in schoolchildren.

The aim of the study is to test the effects of cognitively engaging and simple (low cognitive engagement) classroom-based physical activity breaks on primary schoolchildren's cognitive functions (i.e., response inhibition, lapses of attention, working memory and brain activity), sitting/standing/stepping patterns and on-task behaviour.

It was hypothesised that cognitive functions would improve in both active conditions compared to control, with greater effects observed in the cognitively engaging condition compared to the simple condition. response inhibition and working memory would improve more in the active conditions than the control. Also, it was expected to observe: i) a reduction in children's class time spent sitting and/or an increase in standing/stepping in the active conditions and not in the control; and ii) increased odds of observing children's behaviour as on-task in the active conditions compared to the control group.

Study description

***Study type*:** Interventional

***Procedures*:** Three/four mainstream primary schools will be recruited at convenience from greater Melbourne to participate in the study. Classrooms (Grades 1 and 2) will be randomly assigned to study Arm 1 or Arm 2. One/two of the recruited schools will act as a control group. Teachers in the control group will not be involved in any training sessions and will be asked to continue with the usual school activities. The study Arms are described in Table 1.

Table 1. Description of the intervention.

Study Arm	Name	Description
Arm 1	Cognitively challenging active breaks	Teachers and children assigned to this group will perform cognitively challenging active breaks in their classroom, twice a day for 5-6 weeks. The breaks were designed to last 4-5min. They consisted of physically active games combined with a cognitive task intended to challenge one or more executive functions (i.e., response inhibition, working memory and/or cognitive flexibility) of the participants.

Arm 2	Non-cognitively-challenging (or ‘simple’) active breaks	Teachers and children assigned to this group will perform non-cognitively-challenging active breaks in their classroom, twice a day for 5-6 weeks. The breaks were designed to last 4-5min and had similar intensity, but low cognitive demand compared to Arm 1.
Control	Usual school practice	Teachers and children in this group will not be involved in any training sessions and will be asked to continue with the usual school practice for the entire duration of the study.

***Teacher training*:** All teachers will attend a one-off 20-min training on how to conduct active breaks in the classroom, including a practical demonstration of the breaks. Teachers will be asked to select the active breaks from a specific repertoire of seven activities (different sets for each arm), designed to last between four and five minutes, and to use them to break up children’s prolonged sitting twice a day (between 9:00 am and 11:00 am and between 11:30 am and 1:00 pm) for 5-6 weeks.

***Activities*:** Arm 1 will be required to perform the following activities: i) "My Clock is Late!" – an imitation of a coordination sequence with a time delay between teacher and children [7]; ii) "Silent ball Q&A" – children toss a ball to each other, each time the thrower asks a question to which the catcher has to answer; iii) "Simon says…" – Children perform the actions that are preceded by 'Simon says…', but do nothing in absence of that phrase; iv) "Robot remote control" – a stimulus-response game that requires children remember and perform the appropriate positions/actions associated to each of the teacher’s prompts; v) "One, two, three…star (+ moon, + sun)" – a game that requires children to quickly respond to the stimuli provided by the curator of the game [7]; vi) "Crazy traffic lights" – children are required to move or stop according to the visual signs presented by the teacher, disregarding the inconsistent verbal cues that the teacher will sometimes provide [7]; vii) "Dance off!" – children dance over a music track and freeze their position every time the music stops.

Arm 2 will be required to perform the following activities: i) "Quick fit!" – a simple imitation of a movement sequence; ii) "Silent ball" – children try to toss a light ball to each other without talking, making sounds or dropping the ball; iii) "As if…" – children enact the actions described in the sentences read by the teacher; iv) "Fitness dice" – children perform the activity associated with the result from a dice roll; v) "Over, Under, Around and Through" – children form lines of four/five and go over, under, around and through imaginary or real objects following their leader; vi) "3-speed car" – children pretend to be cars travelling at different speeds as suggested by the teacher; vii) "Let’s dance!" – children dance for the duration of a fun music track.

***Resources*:** Teachers will be provided with an hard copy of a manual, including a description of the activities, specific instructions to be followed for each session, an activity log to record teacher’s daily progress, suggestions on additional resources and equipment that could be used, as well as some equipment (i.e., a light-weight ball, visual cards, action prompts, dices and music).

***Program deliverer(s)*:** one researcher will conduct face-to-face trainings for teachers involved in the trial. Teacher training consists of a one-off 20-min training on how to conduct active breaks in the classroom, including a practical demonstration of the breaks. Teachers will need to select the active breaks from a specific repertoire of seven activities (different sets for each arm), designed to last between four and five minutes, and to use them in the classroom to break up children’s prolonged sitting twice a day (between 9:00 am and 11:00 am and between 11:30 am and 1:00 pm) for 5-6 weeks.

***Adaptations*:** Teachers will be suggested a teaching progression and possible modifications to allow them to tailor the active breaks to the children’s skill levels. Adaptations will be possible by applying at least one of the following: segmentation, modulation of interlimb coordination demand, and adjustments in the ratio between repetition and change. For example, teachers would be able to break down the task to a fewer number of movement types (segmentation), simplify the type of motor tasks performed – e.g., jumping instead of hopping – (modulation of coordination demand) or increase the

number of repetitions of each movement to allow children to have more time to synchronise with the whole group (adjustments in the ratio between repetition and change).

***Adherence*:** The following strategies will allow researchers to control for the fidelity to the program: i) teachers will be asked to complete an activity log to record the number and type of active breaks performed on each trial day; ii) one-on-one interviews will be conducted with teachers at the end of the trial, which will include questions related to fidelity to the program; iii) researchers will incidentally observed teachers' implementation of active breaks during in-class observations of children's on-task behaviour; iv) children's sitting time collected at mid-trial will also be used as a measure of teacher's adherence to the program.

Outcomes

Primary and secondary outcomes of the study and respective measures and timepoints of assessments are presented in Table 2.

Table 2. Study outcomes.

Outcome variable	Measure	Time point
**Primary outcomes**		
Response inhibition	Computer-based Go/No-Go task	Baseline and 5-6 weeks after intervention commencement
Lapses of attention	Exponential component of the ex-Gaussian distribution of reaction time at the Go/No-Go task, used to assess response inhibition.	Baseline and 5-6 weeks after intervention commencement
Brain activity (i.e., changes in oxy-/deoxy-haemoglobin in the prefrontal neocortex)	Artinis® single sensor functional Near-Infrared Spectroscopy (fNIRS), while performing a letter Go/No-Go task (i.e., click for all letters except 'X')	Baseline and 5-6 weeks after intervention commencement
Working memory	iPad-based NIH Toolbox List Sorting Working Memory Test	Baseline and 5-6 weeks after intervention commencement
**Secondary outcomes**		
Sitting/standing/stepping patterns	*Activ*PAL™ inclinometers (activity monitors)	Baseline, 3 weeks, and 5-6 weeks after intervention commencement.[a]
On-task behaviour	Systematic classroom observations.[b]	Baseline, 3 weeks, and 5-6 weeks after intervention commencement
Children's enjoyment of the active breaks	Modified version of the Physical Activity Enjoyment Survey (PACES)	6 weeks after intervention commencement
Perceived physical exertion	Pictorial scale for physical exertion	6 weeks after intervention commencement
Perceived mental effort	Pictorial scale for mental exertion	6 weeks after intervention commencement

[a] At each time point, children will wear the inclinometers for two school days not including physical education or school sports.

[b] Systematic observations of children's behaviour requires a researcher to sit quietly in a corner of the classroom for an hour and to observe six consenting children (selected at random) following the prompts coming from a previously recorded audio file. Each child will be observed for 10 seconds, after which the observed behaviour will noted down (5 seconds). After four consecutive observation intervals the next child will be observed.

Eligibility

***Inclusion* criteria:** Children in Grade 1 and 2 mainstream schools, both males and females, between 6 and 9 years of age.

***Exclusion criteria*:** Having a visual or auditory impairment. This would have not allowed the researchers to measure most of the primary outcomes, as the selected measures are not designed for children with these types of impairments. Having a physical impairment that does not allow children to participate to the breaks would also constitute an exclusion criterion.

Statistical methods / analysis

All data will be processed and analysed using MATLAB (brain activity), Stata 15.0 and R. Linear models will be conducted to investigate the effects of the study condition on children's cognitive functioning (response inhibition, working memory, and brain activity), sitting/stepping, and on-task behaviour, while adjusting for confounders (e.g., sex, age, and classrooms). Brain activity models will also be adjusted for the cognitive performance at the related computer-based cognitive task.

Recruitment

The sample size determination was based on previous studies; a power calculation was not conducted as this was a pilot study. We will recruit around 43 children with typical development per study arm (N = 130 children) and four teachers per study arm (N = 12 teachers).

Identification of potential participants

Prospective participant schools in Melbourne metropolitan area will be identified by using My School database (https://www.myschool.edu.au/). Contact details of all eligible schools will be retrieved on their respective websites. Prospective participants (i.e. children and teachers) will then be invited to participate in the research project via consenting schools.

Initial contact with potential participants

Prospective participant schools will be contacted by phone and briefly provided of a description of the research project aim and objectives. Interested schools will be sent an invitation email with a Plain Language Statement, a consent form, and a copy of ethics approval attached.

Envelopes containing a letter of invitation, an information brochure, a consent form, a withdrawal form, and a demographic information survey will be delivered to prospective participants, via the consenting schools. In addition, schools will be provided with a newsletter article that they can post on their website to promote the research project among prospective participants and their families.

Description of the consent process

Schools, teachers, parents/guardians (on behalf of their children) will read and understand the Plain Language Statement and provide written consent. Schools, teachers, parents/guardians, and children will have the opportunity to ask questions prior to providing written consent. Children for whom we received written consent will be asked to provide verbal assent at the start of their study to confirm their intention to be participate. All participants will have the opportunity to withdraw from the study at any point. Children can choose not to engage in the active breaks or the assessments at any time if they decide they do not want to take part, even where their parent/guardian has provided consent. Prospective participants are free to decline the invitation to participate in the studies without prejudice. Participant's decisions will not affect their relationship with the research team, the schools, and Deakin University in any way.

Use of the data

Collected data will be used for the purpose of this study. Researchers will report the findings in summary of the findings to key stakeholder organisations and participants, in appropriate peer-reviewed publications and in a PhD thesis. As approved by the Ethics committee and consented by the participants, the use of the data is specific to this project. In accordance with the National Health and Medical Research Council (NHMRC) and Deakin University recommendations, the data will be kept for five years from the date of last publication, after which they will be destroyed. Although it could be possible, the use of this data for future research might require additional consent to be sought with participants.

Funding

The study was funded through the Department of Education and Training of Victoria, Australia.

References

1. Tomporowski PD, Pesce C. Exercise, sports, and performance arts benefit cognition via a common process. Psychological Bulletin. 2019/06/14. 2019;145: 929–951. doi:10.1037/bul0000200

2. Cliff DP, Hesketh KD, Vella SA, Hinkley T, Tsiros MD, Ridgers ND, et al. Objectively measured sedentary behaviour and health and development in children and adolescents: Systematic review and meta-analysis. Obesity Reviews. 2016;17: 330–344. doi:10.1111/obr.12371

3. Watson A, Timperio A, Brown H, Best K, Hesketh KD. Effect of classroom-based physical activity interventions on academic and physical activity outcomes: A systematic review and meta-analysis. International Journal of Behavioral Nutrition and Physical Activity. 2017/08/27. 2017;14: 114. doi:10.1186/s12966-017-0569-9

4. Schmidt M, Benzing V, Kamer M. Classroom-based physical activity breaks and children's attention: Cognitive engagement works! Frontiers in Psychology. 2016/10/21. 2016;7: 1474. doi:10.3389/fpsyg.2016.01474

5. Egger F, Benzing V, Conzelmann A, Schmidt M. Boost your brain, while having a break! The effects of long-term cognitively engaging physical activity breaks on children's executive functions and academic achievement. PLoS ONE. 2019;14: e0212482. doi:10.1371/journal.pone.0212482

6. Egger F, Conzelmann A, Schmidt M. The effect of acute cognitively engaging physical activity breaks on children's executive functions: Too much of a good thing? Psychology of Sport and Exercise. 2018;36: 178–186. doi:10.1016/j.psychsport.2018.02.014

7. Tomporowski PD, McCullick BA, Pesce C. Enhancing children's cognition with physical activity games. Human Kinetics; 2015.
